# Supplementary material for: Assessment of Hospital Readiness to Respond to COVID-19 Pandemic in Jordan—A Cross Sectional Study
Source: Int J Environ Res Public Health. 2023 Jan 18;20(3):1798. doi: 10.3390/ijerph20031798 (PMC9913915; doi:10.3390/ijerph20031798)
Supplement: Supplementary file 1 [file ijerph-20-01798-s001.zip › File S1.pdf]

Hospital Readiness Checklist for COVID-19

Hospital Name:

Assessment Date:

ACKNOWLEDGEMENT
This checklist was developed by the World Health Organization (WHO) and was adapted by the USAID Health Service Delivery to the Jordanian context. This adaptation was made possible by the generous support of the American people through the United States Agency for International Development (USAID).

DISCLAIMER
The contents are the responsibility of the USAID Health Service Delivery and do not necessarily reflect the views of USAID or the United States Government.

Hospital Readiness Checklist for COVID-19

Hospitals play a key role in providing health services and essential medical care within health systems. This role is particularly prominent during crises. Many hospitals normally operate at near- surge capacity, and so just a small rise in patient numbers during an emergency can pressure hospitals to work beyond their functional capabilities. The progressive spread of disease during an outbreak can overwhelm hospitals' ability to respond as there are simply too many patients needing medical care at the same time.

Hospitals need to consider their readiness to cope with the influx of patients and increased need for medical services in times of a crisis. Currently, hospitals in Jordan need to prepare for coronavirus 2019 (COVID-19) and a potential influx of patients with severe acute respiratory illness. These hospitals should prioritize and implement actions, specified in their emergency preparedness plans, to identify suspected cases, limit transmission within the facility and provide specialized medical care. This includes activating protocols and procedures in safe physical spaces, emphasizing isolation measures, education and training of personnel in the use of personal protective equipment (PPE), patient management, sample collection and handling, and handling and disposal of hazardous biological waste.[1]

The benefits of an effective hospital-based response include:

- continuity of essential services;
- well-coordinated implementation of priority action;
- clear and accurate internal and external communication;
- swift adaptation to increased demands;
- effective use of scarce resources; and
- a safe environment for health workers.[2]

This checklist is developed by the WHO's Regional Office for the Eastern Mediterranean to support hospital preparedness and response for the management of COVID-19 patients.

USAID Health Service Delivery, in coordination with Jordan's MOH, adapted the checklist to the specific characteristics of Jordanian public hospitals, the legislation governing it, and the national health system and community in which it is based.[3]

The Assessment elements consist of the following areas: leadership and coordination; operational support, logistics and supply management; information and communication; human resources; surge capacity; continuity of essential services; rapid identification; diagnosis; isolation and case management; and infection prevention and control.

Based on the assessment results, the hospital is required to develop an action plan addressing the activities that are not implemented or not completed to ensure the hospital is ready to respond in the advent of different COVID-19 transmission scenarios.

[1] Hospital readiness checklist for COVID-19. Washington (DC): Pan American Health Organization; February 2020.
[2] Hospital readiness checklist for COVID-19. Copenhagen: World Health Organization Regional Office for Europe; 2020.
[3] European Centre for Disease Prevention and Control. Checklist for hospitals preparing for the reception and care of COVID-19 patients. Stockholm: European Centre for Disease Prevention and Control; February 2020.

Hospital Readiness Checklist for COVID-19

Hospital Description

Hospital Name:

Governorate:

**Hospital Category:**	**Teaching Status:**
☐ Ministry of Health	☐ Teaching
☐ Other:	☐ Non-Teaching

Hospital Capacity

**Types of Beds**	**Number of Current Beds** (beds that are routinely equipped and staffed)	**Maximum Surge Capacity**[*]	**Remarks:** Logistics required to meet maximum surge capacity, e.g., where are beds coming from and what space is repurposed.
**General Beds**			
**Emergency Room Beds**			
**Adult ICU Beds**			
**Pediatric ICU Beds**			
**Isolation Beds**			
**Total Number of Beds**			
**Patients Rooms**	**Number of Current Rooms**	**Maximum Surge Capacity**[*]	**Remarks**
**Medical-Surgical**			
**Pediatrics**			
**Isolation**			

[*] The hospital capability to rapidly mobilize resources, unused spaces and beds capacity to meet an increased demand to rapidly expand beyond normal services levels.

USAID FROM THE AMERICAN PEOPLE

وزارة الصحة

World Health Organization Regional Office for the Eastern Mediterranean

Hospital Readiness Checklist for COVID-19		
Annual Occupancy Rate		
ICU Occupancy Rate		
Hospital Mortality Rate		
Hospital Wide Nosocomial Infection Rate / 100 Admissions (2019)		
Personnel		
Specialty	Current Staffing Capacity	Number of additional staff required to respond to "maximum surge capacity"
Anesthesiologists		
Intensivist Physicians		
Internal Medicine Physicians		
Pediatricians		
General Practitioners		
Other Specialties		
Registered Nurses		
Practical Nurses		
Lab Technicians		
Radiology Technicians		
Respiratory Technicians		
Pharmacists		

Hospital Readiness Checklist for COVID-19

Trained Personnel

Topics	Specialty	Number of Trained Personnel	Remarks
**Screening and Triaging**	**Physician**		
	**Nurses**		
	**Other**		
**Clinical Case Management**	**Physician**		
	**Nurses**		
	**Other**		
**Use of Personal Protective Equipment**	**Physician**		
	**Nurses**		
	**Other**		
**Handling and Disposal of Contaminated Waste**	**Physician**		
	**Nurses**		
	**Other**		
**Critical Care and Mechanical Ventilation**	**Physician**		
	**Nurses**		
	**Other**		

Hospital Operational Support

**Number of Adult ICU Ventilators:**	**Number of pediatric ICU Ventilators:**
**Number of Ambulances:**	**Postmortem capacity:**

Available Diagnostic Facilities:

☐ Lab ☐ PCR Test ☐ X-ray ☐ CT scan ☐ MRI

Hospital Readiness Checklist for COVID-19	
**Assessment Details**	
**Date of Assessment:**	
**Name of Assessors:**	**Title of Assessors:**

Hospital Readiness Checklist for COVID-19

No.	Response Readiness Activity	Verification		
**Response Function: Leadership and Coordination**		**Yes**	**No**	**In Process**
1.	Establish/activate Hospital Incident Management team involving representatives from all related departments and units (hospital senior manager, the hospital infection control team, heads of relevant departments/units and an infectious disease expert)			
2.	Designate a secure, easily accessible and well-equipped Hospital Emergency Operations Centre (EOC), with well-functioning means of communication and a dedicated Response Operations Manager.			
3.	Assign roles and responsibilities for the different response functions, with enough trained staff available to ensure operational continuity.			
4.	Develop contingency plans for staffing, logistics, budget, procurement, security and treatment.			
5.	Compile an up-to-date directory of telephone numbers, residence and email addresses of staff and their representative.			
**Response Function: Operational Support, Logistics and Supply Management**		**Yes**	**No**	**In Process**
6.	Coordinate with MOH to ensure the continuous provision of essential medications and supplies (e.g. institutional and central stockpiles, contingency agreements with local suppliers, donations).			
7.	Estimate consumption of essential supplies and pharmaceuticals based on the most likely outbreak scenarios.			
8.	Identify storage facilities for additional stock that meet the storage demands with respect to temperature, humidity, and cold-chain			
9.	Ensure a procedure for the management of work teams, including rest areas, safe transportation and staff well-being.			
10.	Ensure a mechanism for the prompt maintenance and repair of all equipment required for essential services.			
11.	Ensure a procedure for managing ambulances for transportation between hospitals and for the inventory of available vehicles, and a procedure to protect ambulance crew			

No.	Response Readiness Activity	Verification		
	and disinfect ambulance vehicles and equipment after each use.			
12.	Ensure the availability of appropriate back-up arrangements for essential life-lines, including water, electric power and oxygen.			
13.	Solicit the input of hospital security in identifying potential security constraints and optimizing the control of facility access, essential pharmaceutical stocks, patient flow, traffic and parking; seek support from local security forces to augment hospital security, if needed.			
14.	Formulate a postmortem care contingency plan with appropriate partners, for managing an increased need for postmortem care and disposition of deceased patients, and guidelines for the disposal and transport of corpses resulting from the emergency.			
**Response Function: Information and Communication**		**Yes**	**No**	**In Process**
15.	Establish and make available procedures and assign personnel to collect, confirm and validate data and information related to the emergency.			
16.	Provide a standardized form for internal reporting on emergency activities, hospitalizations (including critical care), and incidence of suspected and confirmed cases, clinical situation and deaths.			
17.	Communicate regularly with staff and stakeholders about their roles and responsibilities in managing COVID-19 crisis, clinical triage, patient prioritization and management, hospital epidemiology, reporting requirements and security measures.			
18.	Ensure that all internal protocols, communication lines and standard operating procedures are easily accessible for all staff and information is readily available, concise, targeted and updated regularly (minimize information overload).			
19.	Ensure reliable and sustainable primary and back-up communication systems (e.g. landlines, the internet, mobile devices, two-way radio equipment, and unlisted numbers) and access to updated contact lists.			
**Response Function: Human Resources**		**Yes**	**No**	**In Process**
20.	Adapt human resource management to ensure adequate staff capacity and continuity of operations in response to an			

No.	Response Readiness Activity	Verification		
	increased demand for human resources, while maintaining services identified as essential.			
21.	Prioritize staffing needs by unit or service and distribute personnel accordingly.			
22.	Communicate staffing needs for transmission scenarios to the HAD and MOH.			
23.	Estimate staff absenteeism in advance and monitor it continuously.			
24.	Apply policies and procedures for screening and work restrictions for exposed or ill health care personnel, and develop sick leave policies for health care personnel that are non-punitive, flexible and consistent with public health guidance.			
25.	Inform and train staff who are planned to be reallocated, in accordance with their anticipated roles and responsibilities.			
26.	Identify domestic support measures that could enhance staff flexibility for shift work and longer working hours, and define off-work time for recuperation according to Civil Service bureau instruction			
27.	Ensure the availability of the services of multidisciplinary psychosocial support teams for the staff and patients, including social workers.			
28.	Use occupational health mechanisms that ensure the well-being and safety of personnel during the response to monitor stress-related impacts on staff due to extended working hours.			
29.	Establish a clear policy to monitor and manage staff suspected or confirmed of having COVID-19 or who have been exposed to a confirmed, probable or suspected COVID-19 patient.			
30.	Trained relevant health workers on screening and trigging, clinical case management and infection control.			
**Response Function: Surge Capacity**		**Yes**	**No**	**In Process**
31.	Identify ways of expanding hospital in-patient capacity including physical space (e.g. use of hospital corridors, lobby, parking and other non-essential spaces), staff, supplies and processes.			
32.	Calculate maximum case admission capacity and estimate increase in demand for hospital services during a COVID-19 outbreak.			

No.	Response Readiness Activity	Verification		
33.	Estimate the maximum number of patients' rooms that can be converted into isolation rooms and maximum number of patients that can be cohorted in isolation rooms).			
34.	Coordinate with MOH and local authorities to Identify alternative services as radiology, laboratory, equipment and alternative or secondary patient care sites other than the existing hospital facilities.			
35.	Adapt admission and discharge criteria and prioritize patients and clinical interventions according to available treatment capacity and demand.			
**Response Function: Continuity of Essential Services**		**Yes**	**No**	**In Process**
36.	List all hospital services in priority order and identify nonessential services that could be suspended if necessary, (e.g. canceling specialty consultations, OPD and non-emergency surgical procedures) in order to surge capacities (human and material resources, equipment and physical space).			
37.	Identify the resources (human resources and logistics) needed to ensure continuity of those hospital services identified as essential.			
38.	Determine strategies to maintain services for at-risk patients during the outbreak period (e.g., pregnant patients and those on dialysis) that are unrelated to COVID-19.			
**Response Function: Rapid Identification**		**Yes**	**No**	**In Process**
39.	Train health workers for accurate rapid identification and timely reporting of suspected cases to the corresponding level, in any area of the hospital.			
40.	Have a triage procedure in place in the emergency department with well-equipped triage station at the entrance of the health-care facility, supported by trained staff, focusing on rapid identification of patients with acute respiratory symptoms.			
41.	Develop a system for alternative triage, for example a telephone triage in which patient needs to call first before going to the hospital, to be ready for a more serious transmission scenario, e.g., community transmission.			

No.	Response Readiness Activity	Verification		
**Response Function: Diagnosis**		**Yes**	**No**	**In Process**
42.	Ensure the continuous availability of laboratory and imaging services for diagnosis of COVID-19.			
43.	Develop procedures and train staff in taking samples, proper handling, packaging and transporting them (with biosafety measures in line with transport regulations and requirements) to the designated laboratory.			
44.	Ensure mechanisms for the prompt provision of laboratory data to the physicians, front-line workers and health authorities responsible for clinical management and surveillance.			
45.	Establish a laboratory referral pathway for the identification, confirmation and monitoring of COVID-19.			
**Response Function: Isolation and Case Management**		**Yes**	**No**	**In Process**
46.	Develop and implement hospital strategy, in coordination with local health authorities, for the admission, referral, internal transfer and discharge of patients with severe acute respiratory infections, in line with relevant criteria and protocols.			
47.	Identify, sign and equip areas for the medical care of suspected and confirmed cases in secure and isolated conditions.			
48.	Patients should be placed in an adequately ventilated single rooms (≥ 12 air changes/hour). When single rooms are not available, patients suspected of having COVID-19 should be grouped together. Avoid mixing suspected and confirmed cases.			
49.	Provide guidelines/protocols for the management of suspected or confirmed cases and ensure they are correctly followed.			
50.	Where possible, a team of adequately trained health care workers (HCWs) should be designated to care exclusively for suspected or confirmed cases, to reduce the risk of transmission.			
51.	Maintain a record of all people entering each patient's room, including all staff and visitors.			

No.	Response Readiness Activity	Verification		
52.	Avoid moving and transporting any patient out of their room or area unless it is medically necessary. If transport is required, ensure that procedures for receiving and transferring patients within the hospital and establish the best routes for moving patients			
**Response Function: Infection Prevention and Control**		**Yes**	**No**	**In Process**
53.	Ensure that health care workers (HCWs), patients, and visitors are aware of respiratory and hand hygiene and the prevention of health care-associated infections.			
54.	Ensure availability and proper use protective supplies according to risk stages of clinical posts to ensure correct protection and avoid over protection; establish a registry and tracking system			
55.	Limit visitors to those essential for patient support. Ensure that visitors apply droplet and contact precautions.			
56.	Ensure the facility has infrastructure and procedures for proper hand hygiene, including handwashing, continuous training and supplies.			
57.	Have protocols or procedures available for cleaning and hygiene of clinical areas, including training in the use of decontamination materials.			
58.	Ensure the health facility has dedicated area(s) and protocols for the disinfection and sterilization of biomedical equipment and material devices.			
59.	Ensure the healthcare facility has a protocol and a marked route for the management and final disposal of infectious biological waste, including sharps.			
60.	Ensure strict supervision on the implementation of infection prevention and control measures.			

References:

- Hospital readiness checklist for COVID-19. Washington (DC): Pan American Health Organization; February 2020.

- Hospital readiness checklist for COVID-19. Copenhagen: World Health Organization Regional Office for Europe; 2020.

- European Centre for Disease Prevention and Control. Checklist for hospitals preparing for the reception and care of coronavirus 2019 (COVID-19) patients. Stockholm: European Centre for Disease Prevention and Control; February 2020.

- European Centre for Disease Prevention and Control. Handbook on using the ECDC preparedness checklist tool to strengthen preparedness against communicable disease outbreaks at migrant reception/detention centers. Stockholm: European Centre for Disease Prevention and Control; 2016.

- Office of the Assistant Secretary for Preparedness and Response. COVID-19 healthcare planning checklist. Washington (DC): U.S. Department of Health and Human Services; 2020.

- Centers for Disease Control and Prevention, Office of the Assistant Secretary for Preparedness and Response. Detailed hospital checklist for Ebola preparedness. Washington (DC): U.S. Department of Health and Human Services; 2014.

- Consolidated Ebola virus disease preparedness checklist. Washington (DC): Pan American Health Organization; 2014.

- Hospital emergency response checklist: an all-hazards tool for hospital administrators and emergency managers. Copenhagen: World Health Organization Regional Office for Europe; 2011.
