# Supplementary material for: Assessment of Hospital Readiness to Respond to COVID-19 Pandemic in Jordan—A Cross Sectional Study
Source: Int J Environ Res Public Health. 2023 Jan 18;20(3):1798. doi: 10.3390/ijerph20031798 (PMC9913915; doi:10.3390/ijerph20031798)
Supplement: Supplementary file 1 [file ijerph-20-01798-s001.zip › File S2.pdf]

Hospital Readiness Checklist for COVID-19

Assessment Guide

1. Response Function: Leadership and Coordination			
**Activity**	**Document**	**Interview**	**Observation**
1	• Review TOR of Incident Management Team.<br>• Review sample of the team's meeting minutes.	• Interview team members, ask about their roles and responsibilities.<br>• Validate that they are representative of all related departments	
2		• Interview the dedicated Response Operations Manager on his/her roles and responsibilities.	• Visit the Emergency Operations Center (EOC) to observe accessibility and communication means.
3		• Ask Incident Management Team about the training provided to related staff members to ensure operational continuity.	
4	• Review documented contingency plans.<br>• Verify that the contingency plan contains HR, logistic, budget, security and treatment	• Discuss the process of contingency planning followed by the incident management team.	

**5**	• Check the directory of the up-to-date directory of contact information.	• Ask about the process followed for updating and disseminating the directory to all staff.	• Observe the accessibility of the directory to all staff.

Hospital Readiness Checklist for COVID-19

Assessment Guide

**2. Response Function: Operational Support, Logistics and Supply Management**			
**Activity**	**Document**	**Interview**	**Observation**
**6**	• Review contingency agreements with local suppliers, if available	• Interview Hospital leadership on the coordination process with MOH to ensure supplies.	
**7**	• Review estimations of consumptions rates	• Interview staff responsible for procurement and supplies on process followed to estimate consumption, demand, and zero levels for medical and non-medical supplies.<br>•	
**8**			• Observe facilities identified for additional storage purposes, in terms of: space, temperature, and humidity control.
**9**		• Interview relevant hospital leaders on measures provided to facilitate staff work.	• Observe measures provided for staff well-being including: rest rooms and safe transportation.
**10**	• Review plan and schedules for daily and periodic maintenance of equipment required for essential services.	• Interview staff responsible for equipment management on process followed for maintenance and repair	

Hospital Readiness Checklist for COVID-19

Assessment Guide

2. Response Function: Operational Support, Logistics and Supply Management			
**Activity**	**Document**	**Interview**	**Observation**
11		• Interview relevant staff on procedure followed for managing ambulances, in terms of:<br>▪ Availability of vehicles.<br>▪ Ambulance crew safety.<br>▪ Vehicles disinfection.	▪ Observe the readiness of ambulance vehicles in terms of equipment and supplies availability as well as disinfection.
12	• Review plans for the maintenance of essential life line supplies, including water, oxygen and electricity.	• Interview hospital director and incident management team on arrangements followed for essential life lines back up	• Observe the availability of back up of water supply.<br>• Observe availability and conditions of back up oxygen supply.<br>• Observe availability of back up electric power (Uninterruptable Power Supply)<br>•
13		• Ask hospital security team on the identified security constrains and what actions were taken to augment hospital security.	• Observe the hospital security level and supportive measures taken
14	• Review postmortem contingency plan in case of increased future needs.	• Interview hospital leaders and relevant staff members on guidelines followed for the disposal and transport of corpses resulting form the emergency	

Hospital Readiness Checklist for COVID-19

Assessment Guide

3. Response Function: Information & Communication			
**Activity**	**Document**	**Interview**	**Observation**
**15**		• Interview information management committee and assigned focal person, on procedure followed for the collection, confirmation, and validation of emergency related information.	
**16**	• Review standardized form for internal reporting.		
**17**		• Ask relevant staff members about their roles and responsibilities in crisis management, clinical triaging, patient management, reporting requirements, and security measures.	
**18**	• Review sample of internal protocols, standard operating procedures	• Ask about process followed to ensure that internal policies and procedure are updated and disseminated to staff in a concise manner without overloading them.	• Observe means by which staff members can access up-to-date protocols, policies, and procedures.
**19**			• Observe the availability of primary and back-up communication system in the hospital

4. Response Function: Human Resources			
**Activity**	**Document**	**Interview**	**Observation**
**20**		• Interview human resource department manager on process followed to cope with increased demand in human resources. And how does the hospital ensure adequate staff capacity	
**21**	• Review updated staffing plan per unit or service.	• Interview human resource department manager on the identified top priority staffing needs per unit or service.	
**22**	• Review official letters and submitted requests to cover staffing needs.	• Interview hospital director and human resource department manager, on communications done with health authorities to inform them with staffing needs.	
**23**	• Review the hospital staff absenteeism indicator	• Interview human resource department manager on process followed to estimate and monitor staff absenteeism.	
**24**	• Review policies related to the management of ill or exposed healthcare personnel (Screening, work restrictions).	• Interview staff members on process followed when they are sick.	

4. Response Function: Human Resources			
**Activity**	**Document**	**Interview**	**Observation**
**25**	• Review training topics and attendance list.	• Interview human resources manager and representatives from training and development unit on process followed to train staff members who are to be reallocated.	
**26**		• Interview human resources manager on domestic support measures provided to staff members.	
**27**		• Interview members of the psychosocial support team on their roles and responsibilities to support staff and patients.	
**28**		• Interview human resources manager on the hospital's mechanism to ensure the occupational health of staff and protect them from the stressful impact of extended working hours.	• Observe measures provided to ensure well-being, health and safety of staff members.
**29**	• Review policy related to the management of suspected or confirmed staff of having COVID-19	• Ask the hospital staff about the protocol followed for the management of suspected or confirmed cases among staff members	

4. Response Function: Human Resources			
**Activity**	**Document**	**Interview**	**Observation**
**30**	• Review training material provided on screening, triaging, clinical case management and infection control.<br><br>• Review staff training list, to calculate the percentage of trained staff	• Interview head of training and education unit on the procedure followed to ensure that staff members are trained on COVID-19 management requirements.	

5. Response Function: Surge Capacity			
**Activity**	**Document**	**Interview**	**Observation**
**31**		• Interview Hospital leaders, and incident management teams on ways by which the hospital in-patient capacity can be expanded.	• Observe the ability to expand the hospital in-patient physical space
**32**	• Review calculations of maximum case admission capacity.<br>• Review estimated increase in demand during the outbreak		
**33**		• Interview Hospital leaders, and incident management teams on the estimated maximum number of rooms that can be converted into isolation rooms.<br>• Ask about the maximum number of patients that can be cohorted in isolation rooms.	
**34**		• Interview Hospital leaders, and incident management team on their coordination with MOH to ensure:<br>o Alternative services and equipment.<br>o Alternative or secondary patient care sites.	

5. Response Function: Surge Capacity			
**Activity**	**Document**	**Interview**	**Observation**
**35**	• Review adapted admission and discharge criteria	• Interview relevant staff members in inpatient units and the emergency care unit on how they prioritize patients and interventions according to capacity and demand.	

6. Response Function: Continuity of Essential Services			
**Activity**	**Document**	**Interview**	**Observation**
**36**	• Review list of priority services.<br>• Review list of non-essential services.		
**37**	• Review identified resources needed to ensure continuity of identified essential services. (Human resources and logistics).		
**38**	• Review policy related to the management of high risk COVID-19 patients.	• Interview Hospital leaders, and incident management team on strategies implemented to endure continuity of services for at- risk patients, other than COVID-19 patients. (pregnant patients, and those on dialysis).	

Hospital Readiness Checklist for COVID-19

Assessment Guide

7. Response Function: Rapid Identification			
**Activity**	**Document**	**Interview**	**Observation**
**39**	• Review protocols and training materials related to the rapid identification of COVID-19 patients.	• Interview hospital staff members on the process followed for the reporting of suspected cases to the corresponding level, in any area in the hospital.	
**40**		• Interview staff members in the emergency department on the procedure followed for triaging patients.<br>• Ask about the process followed to rapidly identify patients with acute respiratory symptoms.	• Observe the availability of triage station at the entrance of the facility.
**41**		• Interview hospital leader and the head of emergency department on alternative systems for triaging to ensure pre arrival triaging of patients. Especially during community transmission.	• Observe the availability of alternative triaging system. (e.g. telephone triage)

Hospital Readiness Checklist for COVID-19

Assessment Guide

8. Response Function: Diagnosis			
**Activity**	**Document**	**Interview**	**Observation**
**42**		• Interview laboratory and radiology department heads and ask about process followed to ensure the availability of laboratory and imaging services for diagnosis of COVID-19.	
**43**	• Review procedure followed for sample collection, handling, packaging, and transportation.	• Ask about process followed to ensure relevant staff are trained and competent in sample collection and handling.	• Observe implemented biosafety measures during the collection, and transportation of samples, if possible.
**44**	• Review procedure for COVID-19 laboratory test results reporting to physicians, front-line workers, and health authorities.	• Interview hospital leaders and the head of laboratory department on the reporting process and how they ensure the timeliness of reporting to health authorities.	
**45**		• Interview hospital leaders and the head of laboratory department on the implemented process for referral of laboratory samples. (for identification, confirmation, and monitoring of COVID-19)	

9. Response Function: Isolation & Case Management			
**Activity**	**Document**	**Interview**	**Observation**
**46**	• Review the hospital procedures for admission, referral, internal transfer, and discharge of patient with severe acute respiratory infection.	• Interview hospital leaders and incident management team on the coordination process with local health authorities.	
**47**			• Observe areas designated for the care of suspected and confirmed cases, in terms of:<br>▪ Signage.<br>▪ Security.<br>▪ Isolation conditions.<br>▪ Equipment.
**48**		• Interview relevant staff members on procedure followed to monitor the ventilation and air pressure of patients' isolation rooms.	• Check that suspected patients are separated from confirmed patients in different areas.<br>• Check the ventilation of isolation rooms and air exchange rates.

9. Response Function: Isolation & Case Management			
**Activity**	**Document**	**Interview**	**Observation**
**49**	• Review documented protocols for the management of suspected or confirmed COVID-19 patients.	• Interview clinical staff on the protocols implemented.<br>• Interview physicians and nursing directors on the oversight provided to ensure protocols are followed correctly.	
**50**		• Interview physicians and nursing directors on the availability of exclusively designated staff members who care only for suspected or confirmed COVID-19 cases.	
**51**	• Review sample of records for visitors and staff entering patients' rooms.		
**52**		• Interview clinical staff members about procedure followed to transport patients within the hospital	• Observe identified routes for moving patients

Hospital Readiness Checklist for COVID-19

Assessment Guide

10. Response Function: Infection Prevention & Control			
**Activity**	**Document**	**Interview**	**Observation**
**53**		• Interview hospital leaders on process followed to ensure staff, patients, and visitors' compliance with respiratory and hand hygiene requirements.	• Observe compliance of patients, visitors, and healthcare workers with respiratory and hand hygiene requirements.
**54**	• Review registry of available Personal Protective equipment.	• Interview hospital leaders on the implemented system for the tracking of PPE supplies availability, types, and utilization levels.	
**55**		• Ask about the implemented policy related to patients' visitors.	• Observe visitors of COVID-19 cases in the hospital, and verify the implementation of the hospital's visitor's policy.

10. Response Function: Infection Prevention & Control			
**Activity**	**Document**	**Interview**	**Observation**
**56**			• Observe hand hygiene infrastructure in the hospital:<br>▪ Availability.<br>▪ Location.<br>▪ Supplies (water, soap, hand sanitizers).
**57**	• Review protocols related to cleaning, hygiene, and decontamination of clinical areas.		
**58**	• Review protocols related to disinfection and sterilization of biomedical equipment and material devices.		• Observe areas dedicated to biomedical equipment sterilization and disinfection.

10. Response Function: Infection Prevention & Control			
**Activity**	**Document**	**Interview**	**Observation**
**59**	• Review protocols related to the management and disposal of infectious waste.		• Observe marked routes and areas for final disposal of biological waste, including sharps.
**60**	• Review COVID-19 related infection prevention data and indicators.<br>• Review action plan related to supervision and observation of staff compliance.	• Interview hospital leaders on process followed to ensure strict compliance with infection prevention and control measures.	• Observe staff behavior and compliance with PPE use.
