# Supplementary material for: Assessment of Hospital Readiness to Respond to COVID-19 Pandemic in Jordan—A Cross Sectional Study
Source: Int J Environ Res Public Health. 2023 Jan 18;20(3):1798. doi: 10.3390/ijerph20031798 (PMC9913915; doi:10.3390/ijerph20031798)
Supplement: Supplementary file 1 [file ijerph-20-01798-s001.zip › File S3.pdf]

Name Hospital Readiness Assessment for COVID-19

Assessment Agenda

Day-Date		
**Surveyor**	**Surveyor I**	**Surveyor II**
**Assigned Response Functions**	**1. Leadership & Coordination.**<br>**2. Operational Support. Logistics and Supply Management.**<br>**3. Information Communication.**<br>**4. Human Resources.**<br>**5. Continuity of Essential Services.**	**6. Surge Capacity**<br>**7. Rapid Identification.**<br>**8. Diagnosis**<br>**9. Isolation, Case Management**<br>**10. Infection Prevention and Control**
**9:00am-9:15am**	**Introductory Meeting -Overview of the Agenda**	
**9:15am-10:30am**	**Document Review Response Functions (1-5)**	**Document Review Response Functions (6-10)**
**10:30am-11:45am**	**Teams meeting & Interviews**<br><br>**Response Functions (1-5)**	**Teams meeting & Interviews**<br><br>**Response Functions (6-10)**
**11:45am-12:30pm**	**Break**	
**12:30pm-2:30 pm**	**Facility Tour:**<br>• Emergency Operations Centre (EOC).<br>• Supplies & Consumables Storage Areas.<br>• Staff Rest Rooms<br>• Ambulance Vehicles.<br>• Back Up Oxygen Supplies.<br>• Back Up water Supplies.<br>• Back Up Electrical Supplies.<br>• Hospital Entrance.<br>• Facility spaces(corridor-lobby-parking)<br>•	**Facility Tour**<br>• Patient Care Areas.<br>• Laboratory Department.<br>• Radiology Department.<br>• Triage station.<br>• Isolation Rooms.<br>• Waste Containers.<br>• Washing facilities.<br>• Biomedical Equipment Sterilization-Disinfections Areas.<br>• Biological Waste Disposal Areas
**2:30pm-3:00pm**	**Assessment team Meeting- findings integration**	
**3:00pm – 3:30pm**	**Debriefing and Wrap-up**	
