# Supplementary material for: Assessment of Hospital Readiness to Respond to COVID-19 Pandemic in Jordan—A Cross Sectional Study
Source: Int J Environ Res Public Health. 2023 Jan 18;20(3):1798. doi: 10.3390/ijerph20031798 (PMC9913915; doi:10.3390/ijerph20031798)
Supplement: Supplementary file 1 [file ijerph-20-01798-s001.zip › File S4.pdf]

Hospital Readiness Assessment for COVID-19

Required Documents List

#	Required Documents
1	**Incident Management Team TOR**
2	**Hospital COVID-19 Contingency Plans:** (HR, logistic, budget, security and treatment)
3	**Hospital's directory of the up-to-date staff contact information**
4	**Contingency agreements with local suppliers for the provision of essential medications and supplies.**
5	**Estimations of consumptions and demand rates of medical and non-medical supplies**
6	**Inventory list of available Personal Protective Equipment**
7	**Plan and schedules for daily and periodic maintenance of equipment required for essential services.**
8	**plans for the maintenance of essential life line supplies, including water, oxygen and electricity**
9	**Post-mortem contingency plan in case of increased future needs.**
10	**Standardized forms and templates for internal reporting.**
11	**Sample of internal staff standard operating procedures**
12	**Updated staffing plan per unit or service**
13	**official letters and communications of staffing needs requests**
14	**Hospital staff absenteeism indicator data and results**
15	**Policies related to the management of ill or exposed healthcare personnel (Screening, work restrictions).**
16	**Hospital staff COVID-19 related training materials and training attendance lists, related to:**<br>• Human resources updated policies and procedures.<br>• Screening, triaging, clinical case management and infection control protocols.<br>• management of high risk COVID-19 patients.

	• Protocols for the rapid identification of COVID-19 patients.<br>• protocols for the management of suspected or confirmed COVID-19 patients.<br>• PCR sample collection, handling, packaging, and transportation.<br>• procedures for admission, referral, internal transfer, and discharge of patient with severe acute respiratory infection.<br>• procedure for COVID-19 laboratory test results reporting to physicians, front-line workers, and health authorities.<br>• Protocols related to cleaning, hygiene, and decontamination of clinical areas.<br>• Protocols related to disinfection and sterilization of biomedical equipment and material devices.
**17**	**Hospital's policy related to the management of suspected or confirmed staff of having COVID-19**
**18**	**Maximum case admission capacity, and estimations of increased demands**
**19**	**COVID-19 admission discharge criteria.**
**20**	**List of Priority services, and non-essential services.**
**21**	**Policy related to visitor's management**
**22**	**Policy related to the management of high risk COVID-19 patients.**
**23**	**Protocols related to the rapid identification of COVID-19 patients.**
**24**	**Procedure followed for sample collection, handling, packaging, and transportation**
**25**	**Procedure for COVID-19 laboratory test results reporting to physicians, front-line workers, and health authorities.**
**26**	**Procedures for admission, referral, internal transfer, and discharge of patient with severe acute respiratory infection.**
**27**	**Protocols for the management of suspected or confirmed COVID-19 patients.**
**28**	**Protocols related to cleaning, hygiene, and decontamination of clinical areas.**
**29**	**Protocols related to disinfection and sterilization of biomedical equipment and material devices.**
